# Supplementary material for: A cortical surface template for human neuroscience
Source: Nat Methods. 2024 Jul 16;21(9):1736–42. doi: 10.1038/s41592-024-02346-y (PMC11399089; doi:10.1038/s41592-024-02346-y)
Supplement: Supplementary file 1 — Supplementary Note (Supplementary Methods and Supplementary Results) and Supplementary Figs. 1–6. [file 41592_2024_2346_MOESM1_ESM.pdf]

nature methods

Supplementary information https://doi.org/10.1038/s41592-024-02346-y

A cortical surface template for human neuroscience

In the format provided by the authors and unedited

Supplementary Note

In the main Results and its replications (Fig. 2, Extended Data Figs. 2–6, Supplementary Figs. 1–5), we demonstrated the advantages of the onavg template for various MVPA analyses and the robustness of these advantages across a wide range of conditions. We performed additional analyses on functional connectivity, contrast maps, and individual differences based on the Human Connectome Project (HCP) dataset to showcase the general applicability of the onavg template.

For the analyses of functional connectivity and contrast maps, we used the 3 T resting-state and task fMRI data of the HCP dataset, respectively. Both analyses were based on 888 participants who had complete 3 T fMRI data. For the analysis of individual differences, we used the 7 T data with movie-watching, and it included 178 participants, which partly overlap with the 888 3 T participants (149 overlapped participants). One participant (221319) was excluded from the 7 T data analysis due to data quality (including the participant does not change any conclusions).

Demographics of validation data

The validation data are also datasets that have been previously collected. The *Forrest* dataset has 15 participants (6 females and 9 males). Among them, 5 were 20–25 yo, 3 were 25–30 yo, 6 were 30–35 yo, and 1 was 35–40 yo. The *Raiders* dataset has 23 participants (12 females and 11 males), with an age range of 22–31. The mean age ± standard deviation was 27.26 ± 2.36 yo. The *Budapest* dataset has 21 participants (11 females and 10 males), with an age range of 22–31. The mean age ± standard deviation was 27.29 ± 2.35 yo.

The validation data used in some Extended Data Figures were from the Human Connectome Project (HCP) dataset. The 888 participants of the 3 T data were 469 females and 419 males. The age range was 22–37, and the mean age ±

standard deviation was 28.65 ± 3.72 yo. The 176 participants of the 7 T data were 106 females and 70 males. The age range was 22–36, and the mean age ± standard deviation was 29.36 ± 3.30 yo.

Resting-state functional connectivity

For the resting-state functional connectivity analysis, we computed the connectivity between each vertex and its neighbors, and averaged it across neighbors, scans, and participants. We refer to this average value as the local connectivity of the vertex. Local connectivity is known to be biased by the geometric shape of the cortex [63]. This is because vertices nearby are largely resampled from the same group of voxels, and their time series are linear combinations of the same set of basis. This issue is more severe when the vertices are closer, as in this case their weights of the linear combination are more similar to each other.

We performed the analysis using both synthesized noise and real fMRI data. The real fMRI data are the 4 resting-state fMRI scans of the 3 T HCP dataset, and the synthesized data are volumes of similar shape and resolution, except that the numbers were randomly drawn from a standard Gaussian distribution. This allows us to separate the effects of cortical resampling from the effects of MRI's point spread function and the "true" local connectivity. We found that the onavg template reduced the bias of local functional connectivity for both synthesized noise and real fMRI data (Extended Data Fig. 7).

Functional contrast maps

The 3 T task fMRI data of the HCP dataset comprises 7 different tasks, from which 47 unique contrasts can be computed [64]. Some contrasts, such as "FACES-SHAPES" and "SHAPES-FACES," are actually the same contrast with flipped signs,

and in such cases we only used one of them. We computed each contrast in each participant's native space, and resampled the contrast to different template spaces. The transform used for resampling was the sum of two barycentric transforms, that is, the transform from the native space to the template space, and the transpose of the transform from the template space to the native space. This ensures that every vertex in the native space is used in the resampling process. After deriving the transform, we normalized it so that the sum of each column equals one. This normalization ensures that the resampled data (template space) has the same scale as the original data (native space).

We computed the ISC of contrast maps in a similar manner as the RSA-ISC analysis. That is, for each participant, we computed the correlation between the participant's own map and the average of others' maps. The only difference was that this analysis was based on the contrast map of the entire cortex, whereas the RSA-ISC analysis was based on the vectorized RDM of each searchlight. Similar to the RSA-ISC analysis, we also estimated how this correlation would change with different amounts of data. When we compared the ISC across template spaces, we averaged the ISC across the 47 contrasts, and tested whether the average ISC differed between onavg and other template spaces (n = 888). For each comparison (onavg vs. fsavg, onavg vs. fslr), we also performed a permutation test with 100,000 repetitions, which allowed us to compare the actual differences with the corresponding null distributions (Extended Data Fig. 8).

Individual differences in functional architecture

We used neural responses to the movie from 178 participants of the HCP 7 T dataset for this analysis. We used 89 participants' data (training data) to build a functional template and performed the analysis using the remaining 88 participants

(test data; excluding 1 participant for data quality). Specifically, we modeled the neural responses to the movie of different participants as the same functional template transformed with individualized transforms. After applying the individualized transform to the template, we converted the transformed functional template into a tuning matrix, which depicts the neural tuning of the specific participant [36]. The tuning matrix was designed so that (a) vertices (or individuals) that are more different in response time series are also more different in their tuning profiles (i.e., columns of the tuning matrix), (b) the same tuning matrix can be estimated from responses to different stimuli. This allowed us to compute two estimates of the tuning matrix for each participant by dividing the participant's movie data into two halves and estimating the tuning matrix from each half.

For each pair of participants, we computed the similarities between their tuning matrix estimates. The similarity was always based on tuning matrices estimated from different halves of the movie, that is, the first half of the movie for one participant, and the second half of the movie for the other. Therefore, for each pair of participants, we had two tuning matrix similarities, and we used the average of them as the between-participant similarity for this pair of participants. Similarly, for each participant, we computed a within-participant similarity based on the two tuning matrix estimates of the participant.

After looping through all participants and participant pairs, for each participant, we had 1 within-participant similarity and 87 between-participant similarities. We computed the distinctiveness of the participant as the difference between the within-participant similarity and average between-participant similarity, divided by the standard deviation of between-participant similarity [36]. In other words, the distinctiveness measures how far away the within-participant similarity was from the

distribution of between-participant similarities, and higher distinctiveness means the differences between the participant and others are more prominent.

We repeated the analysis with different surface templates, and thus each participant had three distinctiveness values, one for each template. We compared the distinctiveness between onavg and the other two templates. We also repeated the analysis with different amounts of data, that is, 7 minutes, 14 minutes, …, up to the entire movie data (60.91 minutes). The onavg template consistently demonstrated higher distinctiveness than other templates across different amounts of data (Extended Data Fig. 9).

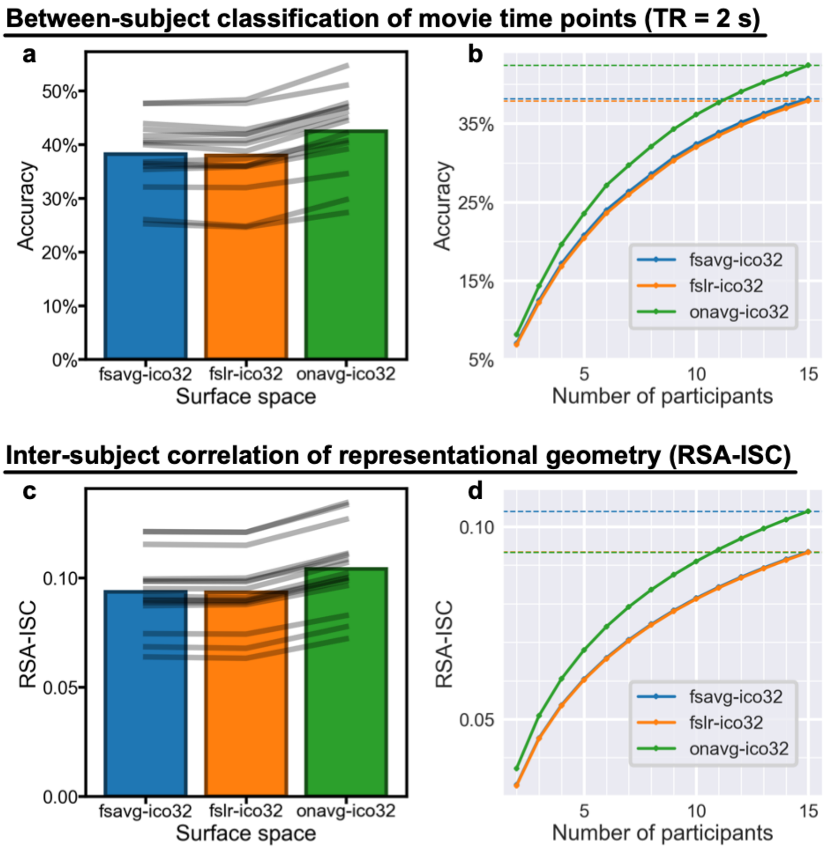

Supplementary Fig. 1. Better cortical sampling improves MVPA results (Procrustes hyperalignment). a) The between-subject classification accuracy of movie time points based on fsavg, fslr, and onavg. Bars denote the average accuracy across all 15 participants. Gray lines denote the accuracies of individual participants. b) Classification accuracy as a function of the amount of data (the number of participants). Dashed horizontal lines denote accuracies when n = 15. c) RSA-ISC, computed as the correlation between one participant's RDM and the average of others', based on fsavg, fslr, and onavg. Bars denote the average RSA-ISC across 15 participants, and gray lines denote those of individual participants. d) RSA-ISC as a function of the amount of data. Dashed horizontal lines denote RSA-ISC when n = 15.

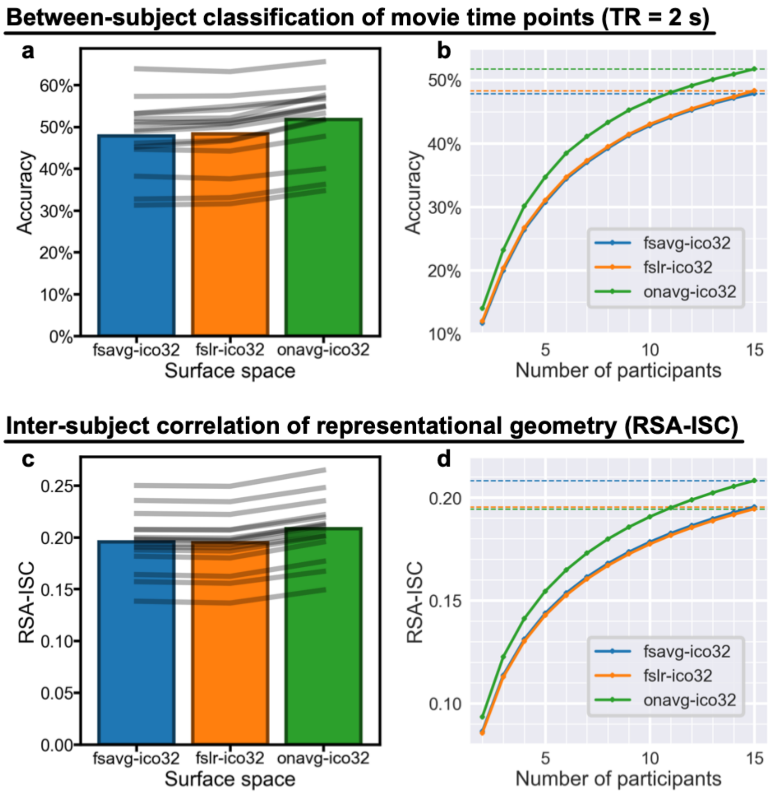

Supplementary Fig. 2. Better cortical sampling improves MVPA results (warp hyperalignment). a) The between-subject classification accuracy of movie time points based on fsavg, fslr, and onavg. Bars denote the average accuracy across all 15 participants. Gray lines denote the accuracies of individual participants. b) Classification accuracy as a function of the amount of data (the number of participants). Dashed horizontal lines denote accuracies when n = 15. c) RSA-ISC, computed as the correlation between one participant's RDM and the average of others', based on fsavg, fslr, and onavg. Bars denote the average RSA-ISC across 15 participants, and gray lines denote those of individual participants. d) RSA-ISC as a function of the amount of data. Dashed horizontal lines denote RSA-ISC when n = 15.

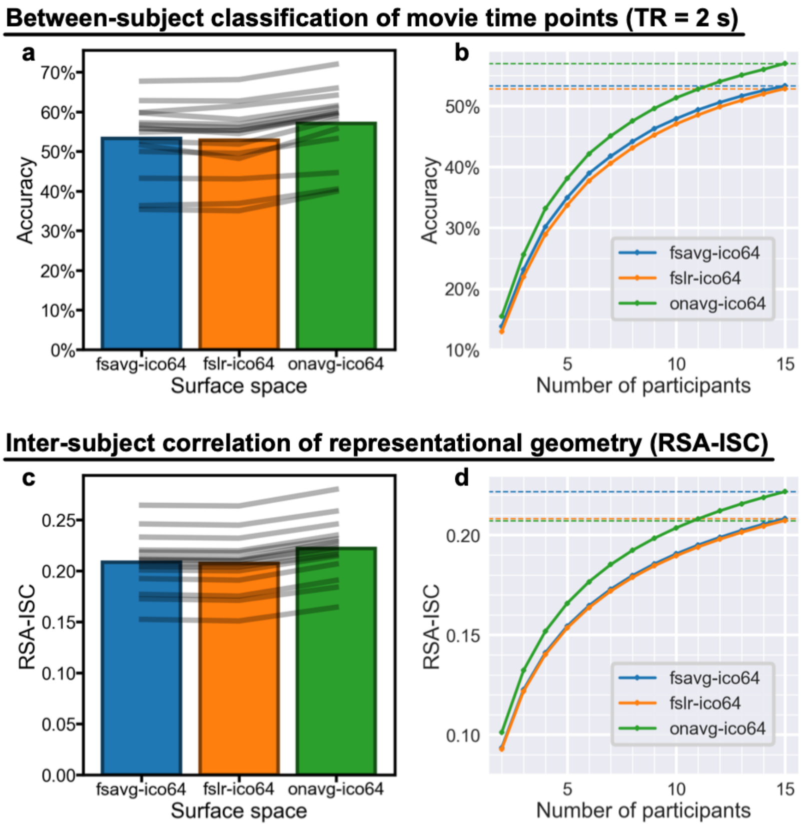

Supplementary Fig. 3. Better cortical sampling improves MVPA results (warp hyperalignment, ico64 resolution). a) The between-subject classification accuracy of movie time points based on fsavg, fslr, and onavg. Bars denote the average accuracy across all 15 participants. Gray lines denote the accuracies of individual participants. b) Classification accuracy as a function of the amount of data (the number of participants). Dashed horizontal lines denote accuracies when *n* = 15. c) RSA-ISC, computed as the correlation between one participant's RDM and the average of others', based on fsavg, fslr, and onavg. Bars denote the average RSA-ISC across 15 participants, and gray lines denote those of individual participants. d) RSA-ISC as a function of the amount of data. Dashed horizontal lines denote RSA-ISC when *n* = 15.

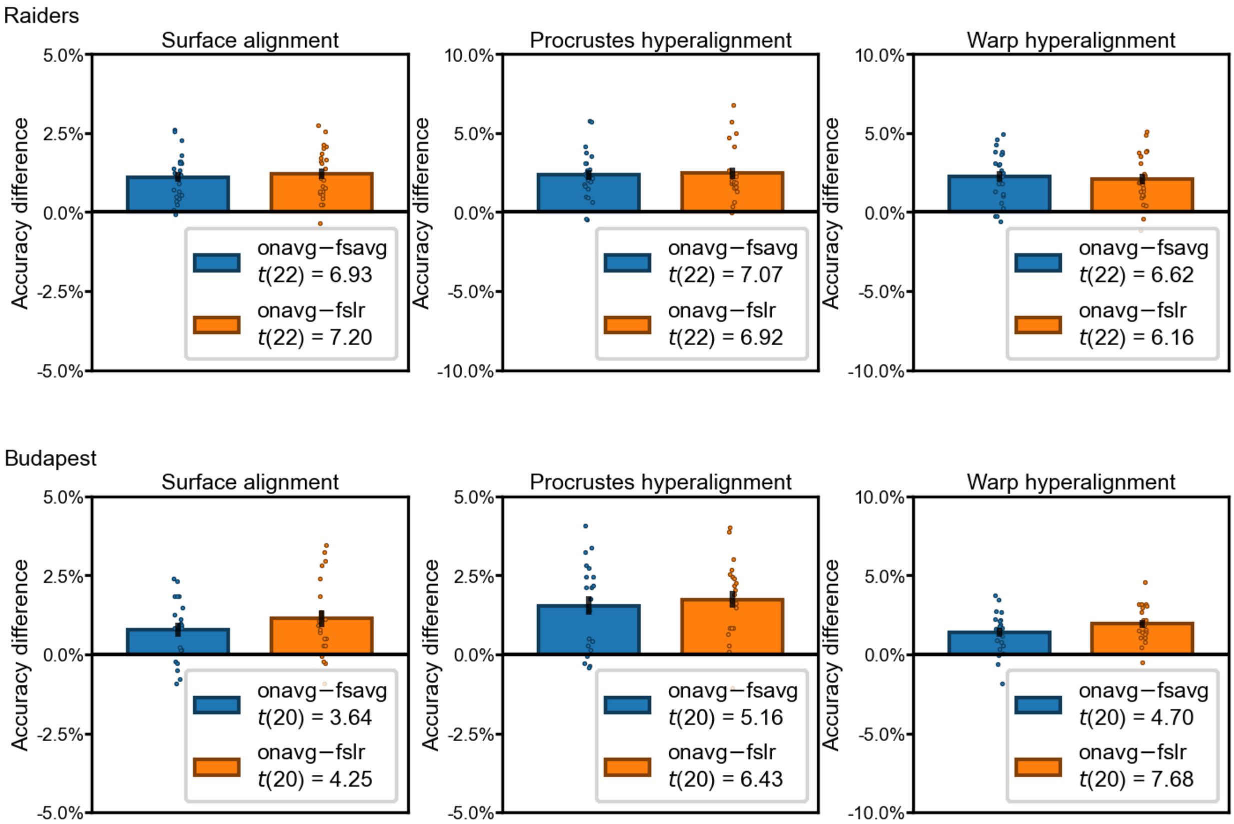

Supplementary Fig. 4. Statistical comparisons of classification accuracy. The difference of classification accuracy between onavg and fsavg, and between onavg and fslr, across two datasets and three alignment methods. The difference was tested using paired t-test based on n = 23 and 21 participants for the *Raiders* and *Budapest* datasets, respectively. Each individual dot represents the classification accuracy difference of a single participant. The dots were slightly jittered horizontally to increase visibility. Error bars denote mean values ± SEM.

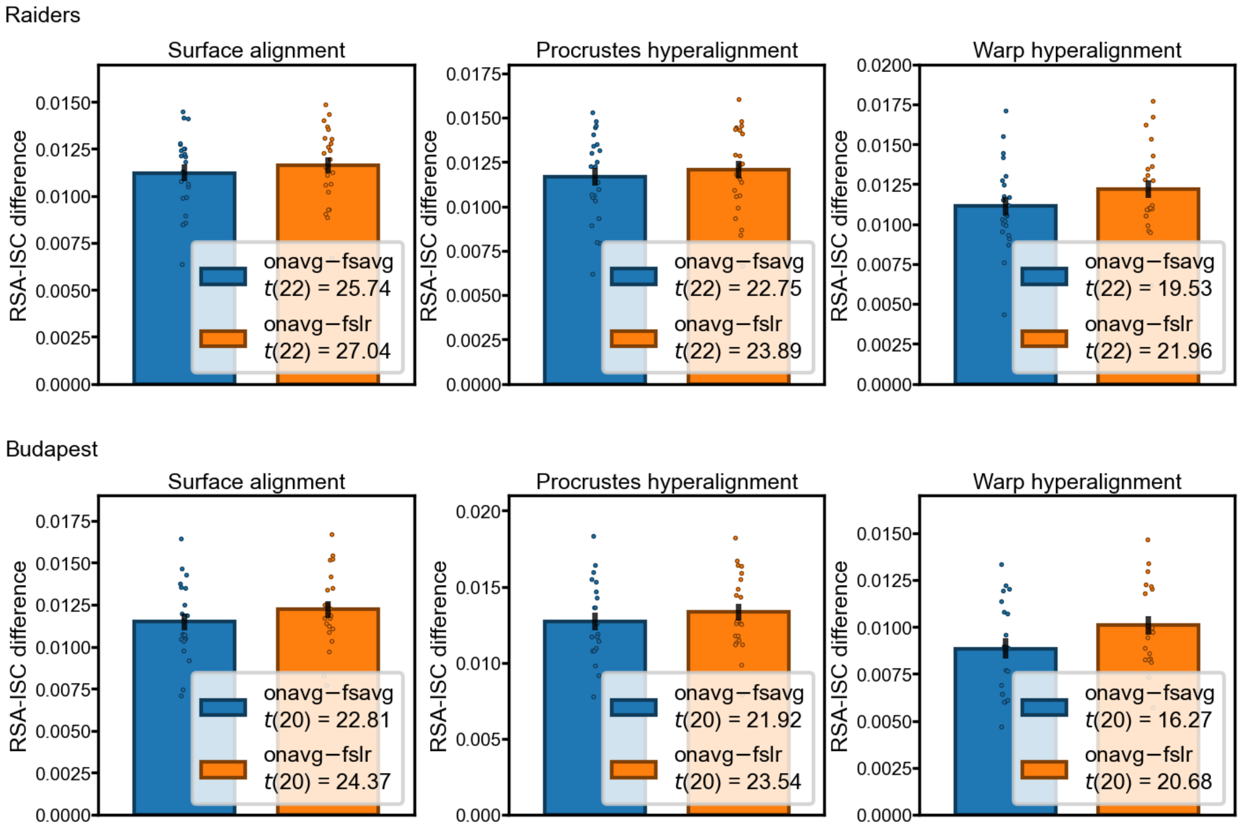

Supplementary Fig. 5. Statistical comparisons of RSA-ISC. The difference of RSA-ISC between onavg and fsavg, and between onavg and fslr, across two datasets and three alignment methods. The difference was tested using paired t-test based on n = 23 and 21 participants for the *Raiders* and *Budapest* datasets, respectively. Each individual dot represents the RSA-ISC difference of a single participant (averaged across all searchlights). The dots were slightly jittered horizontally to increase visibility. Error bars denote mean values ± SEM.

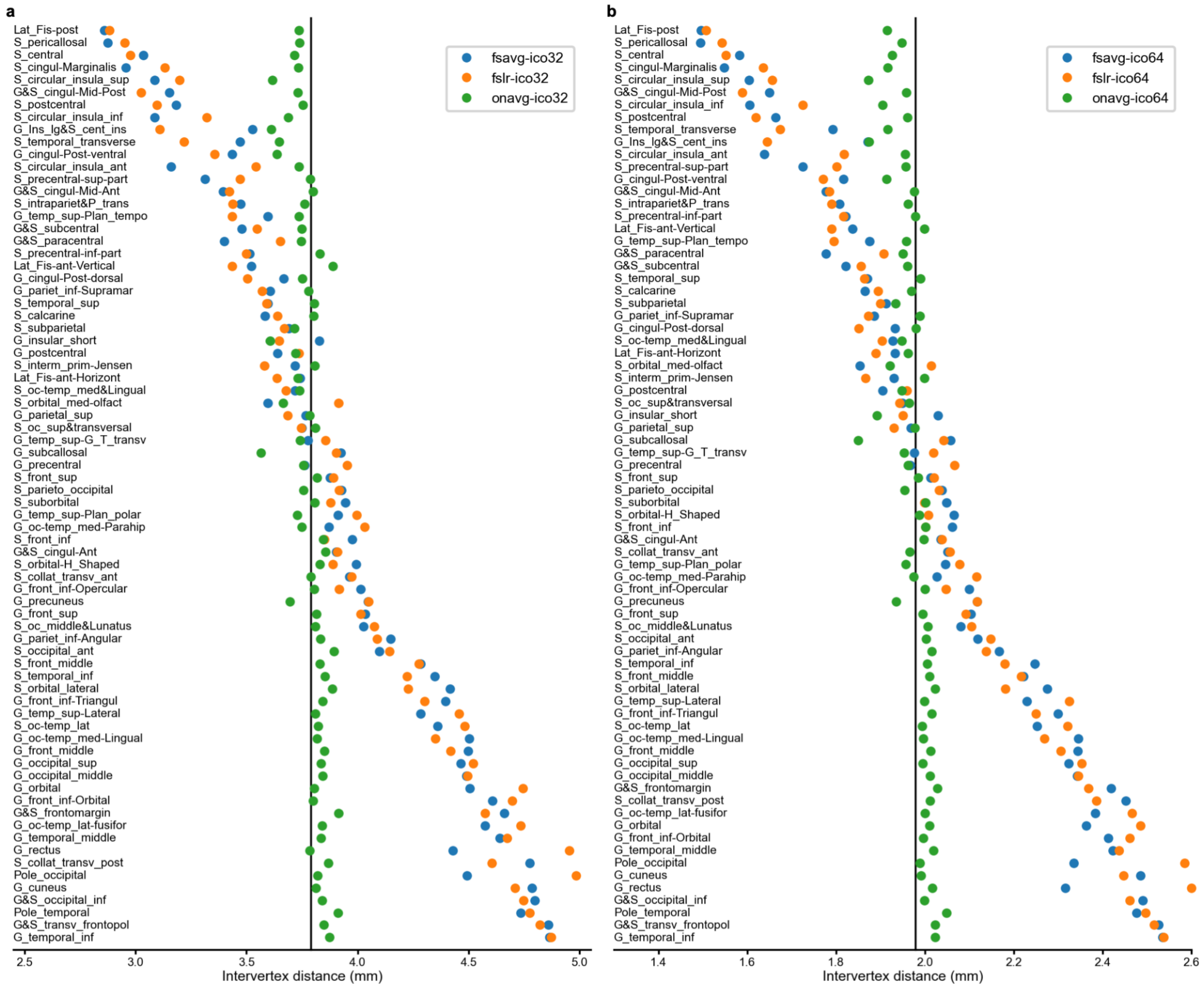

Supplementary Fig. 6. Inter-vertex distance by brain region. a) Inter-vertex distance by brain region for fsavg, fslr, and onavg, based on the ico32 resolution (approximately 4 mm). b) Inter-vertex distance by brain region based on the ico64 resolution (approximately 2 mm).

Supplementary References

64. Barch, D. M. *et al.* Function in the human connectome: Task-fMRI and individual differences in behavior. *NeuroImage* **80**, 169–189 (2013).
