# Supplementary material for: A cortical surface template for human neuroscience
Source: Nat Methods. 2024 Jul 16;21(9):1736–42. doi: 10.1038/s41592-024-02346-y (PMC11399089; doi:10.1038/s41592-024-02346-y)
Supplement: Supplementary file 2 — Reporting Summary [file 41592_2024_2346_MOESM2_ESM.pdf]

nature portfolio

Corresponding author(s): Ma Feilong, James V. Haxby

Last updated by author(s): Mar 29, 2024

Reporting Summary

Nature Portfolio wishes to improve the reproducibility of the work that we publish. This form provides structure for consistency and transparency in reporting. For further information on Nature Portfolio policies, see our Editorial Policies and the Editorial Policy Checklist.

Statistics

For all statistical analyses, confirm that the following items are present in the figure legend, table legend, main text, or Methods section.

n/a	Confirmed	
☐	☒	The exact sample size (*n*) for each experimental group/condition, given as a discrete number and unit of measurement
☐	☒	A statement on whether measurements were taken from distinct samples or whether the same sample was measured repeatedly
☐	☒	The statistical test(s) used AND whether they are one- or two-sided<br>*Only common tests should be described solely by name; describe more complex techniques in the Methods section.*
☒	☐	A description of all covariates tested
☒	☐	A description of any assumptions or corrections, such as tests of normality and adjustment for multiple comparisons
☐	☒	A full description of the statistical parameters including central tendency (e.g. means) or other basic estimates (e.g. regression coefficient) AND variation (e.g. standard deviation) or associated estimates of uncertainty (e.g. confidence intervals)
☐	☒	For null hypothesis testing, the test statistic (e.g. *F*, *t*, *r*) with confidence intervals, effect sizes, degrees of freedom and *P* value noted<br>*Give P values as exact values whenever suitable.*
☒	☐	For Bayesian analysis, information on the choice of priors and Markov chain Monte Carlo settings
☒	☐	For hierarchical and complex designs, identification of the appropriate level for tests and full reporting of outcomes
☐	☒	Estimates of effect sizes (e.g. Cohen's *d*, Pearson's *r*), indicating how they were calculated

Our web collection on statistics for biologists contains articles on many of the points above.

Software and code

Policy information about availability of computer code

Data collection	Data were downloaded from OpenNeuro (https://openneuro.org/) using DataLad 0.17.2 (https://www.datalad.org/).
Data analysis	The analysis was performed in a Python 3.8.12 environment with standard scientific computing packages and libraries, including numpy 1.16.4, scipy 1.3.0, pandas 0.24.2, joblib 0.13.2, matplotlib 3.1.0, and seaborn 0.9.0. The data analysis was performed using the Discovery HPC cluster of Dartmouth College. Cortical surface reconstruction was performed using FreeSurfer (freesurfer-Linux-centos6_x86_64-stable-pub-v6.0.1-f53a55a) that was shipped within the fMRIPrep docker image (21.0.1).<br>The code used to create the onavg template and to perform the benchmarking analyses are available through GitHub (https://feilong.github.io/tpl-onavg/). This GitHub Pages website also contains detailed tutorials on how to use the onavg template and how to transform data between onavg and other templates. The code, along with other information provided in the website, is also archived as a Zenodo repository (https://zenodo.org/records/10535655).

For manuscripts utilizing custom algorithms or software that are central to the research but not yet described in published literature, software must be made available to editors and reviewers. We strongly encourage code deposition in a community repository (e.g. GitHub). See the Nature Portfolio guidelines for submitting code & software for further information.

Data

Policy information about availability of data

All manuscripts must include a data availability statement. This statement should provide the following information, where applicable:

- Accession codes, unique identifiers, or web links for publicly available datasets
- A description of any restrictions on data availability
- For clinical datasets or third party data, please ensure that the statement adheres to our policy

The onavg template is available at TemplateFlow, the standard repository for brain templates, as a DataLad dataset (https://github.com/templateflow/tpl-onavg). Additional group statistics based on the 1,031 participants, such as average maps of sulcal depth, curvature, and vertex area, are available through GIN as a DataLad dataset (https://gin.g-node.org/neuroboros/core).
The data of the 1,031 participants that were used to create the onavg template are available through OpenNeuro (https://openneuro.org/) as ds000031, ds000201, ds000221, ds000224, ds000256, ds001233, ds001399, ds001499, ds001597, ds002278, ds002320, ds002330, ds002345, ds002382, ds002634, ds002685, ds002702, ds002737, ds002766, ds002799, ds003242, ds003452, ds003465, ds003499, ds003653, ds003701, ds003745, ds003752, ds003787, and ds003849. The Forrest dataset is available through OpenNeuro as ds000113, and it can also be accessed through the studyforrest website (https://www.studyforrest.org/). The Budapest dataset is available through OpenNeuro as ds003017. The Human Connectome Project data is available through ConnectomeDB (https://db.humanconnectome.org/).

Human research participants

Policy information about studies involving human research participants and Sex and Gender in Research.

Reporting on sex and gender	Out of the 30 OpenNeuro datasets used to create the onavg cortical surface template, 25 reported demographics information. Two datasets (ds000221 and ds000224) reported gender; one dataset (ds001597) did not report sex or gender; the remaining 22 datasets (ds000201, ds000256, ds001233, ds001399, ds001499, ds002278, ds002330, ds002345, ds002382, ds002685, ds002702, ds002737, ds002799, ds003242, ds003452, ds003465, ds003499, ds003653, ds003701, ds003745, ds003787, ds003849) reported sex. For each spatial resolution, we created a single surface template across sex and gender.
Population characteristics	Participants were primarily young adults (mean age = 28.42 years, std = 14.57 years). 471 were female, 408 were male, and 2 were non-binary. Out of the 1,031 participants, age was unknown for 264, and sex/gender was unknown for 150. Participants had no visible lesion or structural abnormality.
Recruitment	Participants were recruited by the original researchers of the 30 OpenNeuro datasets.
Ethics oversight	This study re-analyzes openly available datasets. These datasets were respectively collected by other researchers and approved by their institutions. Details on each dataset can be found on OpenNeuro (e.g., https://openneuro.org/datasets/ds000031), and additional information is available in the "References and Links" section of each dataset.

Note that full information on the approval of the study protocol must also be provided in the manuscript.

Field-specific reporting

Please select the one below that is the best fit for your research. If you are not sure, read the appropriate sections before making your selection.

☐ Life sciences ☒ Behavioural & social sciences ☐ Ecological, evolutionary & environmental sciences

For a reference copy of the document with all sections, see nature.com/documents/nr-reporting-summary-flat.pdf

Behavioural & social sciences study design

All studies must disclose on these points even when the disclosure is negative.

Study description	We used high-quality structural scans of 1,031 participants to create a cortical surface template that evenly samples the cerebral cortex.
Research sample	To maximize the quality of the template, we used as much openly available high-quality data as possible when we started the project in Feb 2022. Specifically, we used OpenNeuro datasets that have a CC0 or PDDL license, and ensured that: (a) each participant has at least one T1w scan and one T2w scan, (b) the scans cover the entire cerebral cortex; (c) the spatial resolution is 1 mm or less in all directions, and (d) the data have no major quality issues. The sample is representative of the population of all neuroimaging research participants, and the demographics of the sample match those of other datasets (see Supplementary Note). The rationale for choosing this sample is that both larger sample size and better data quality will increase the quality of the final template. Therefore, we tried to make the sample size as large as possible while making sure that we only used data that had passed the quality check. In other words, the procedure was designed to maximizing the template quality by achieving a balance between sample size and data quality.

Sampling strategy	The OpenNeuro datasets afford convenience sampling of all participants of neuroimaging research. No sample size calculation was performed, and we aimed to use as much data that pass the quality check as possible. We ended up with a sample size of 1,031 participants, which is 25 times more than previous samples used to build cortical surface templates. Therefore, it is reasonable to believe that the sample size is sufficient for the current study.
Data collection	No new data was collected for the current study. The 30 OpenNeuro datasets were collected by their original researchers, and therefore these researchers were blinded to the hypothesis of the current study. The structural scans of these datasets were acquired using 3 T MRI scanners.
Timing	Not applicable because we did not collect new data or recruit participants for this study. Not all OpenNeuro datasets have clearly documented start and stop dates of data collection. However, it is unlikely for the structural scans to be affected by the timing of data collection.
Data exclusions	We excluded participants whose cortical reconstruction could not be properly performed. This is usually caused by quality issues of the structural scans and accompanied by a prolonged "mris_fix_topology" step of FreeSurfer.
Non-participation	Not applicable because we did not collect new data or recruit participants for this study.
Randomization	We used leave-one-participant-out cross-validation for the MVPA analyses described below, which does not involve randomization.

Reporting for specific materials, systems and methods

We require information from authors about some types of materials, experimental systems and methods used in many studies. Here, indicate whether each material, system or method listed is relevant to your study. If you are not sure if a list item applies to your research, read the appropriate section before selecting a response.

Materials & experimental systems

n/a	Involved in the study
☒	☐ Antibodies
☒	☐ Eukaryotic cell lines
☒	☐ Palaeontology and archaeology
☒	☐ Animals and other organisms
☒	☐ Clinical data
☒	☐ Dual use research of concern

Methods

n/a	Involved in the study
☒	☐ ChIP-seq
☒	☐ Flow cytometry
☐	☒ MRI-based neuroimaging

Magnetic resonance imaging

Experimental design

Design type	We used the naturalistic movie viewing data of the StudyForrest dataset.<br>In Supplementary Information, we also used the Raiders dataset, Budapest dataset, and the Human Connectome Project (HCP) dataset.
Design specifications	Participants watched the feature movie Forrest Gump in the scanner. The movie was divided into 8 parts, and the length of the runs were adjusted accordingly. See Hanke et al. (2016, 10.1038/sdata.2016.92) for details.<br>The two other movie datasets (Raiders, Budapest) had similar designs.<br>The HCP data we used including 3 T resting-state fMRI data, 3 T task fMRI data, and 7 T movie-watching fMRI data. The data are described in detail in its documentation website (https://www.humanconnectome.org/study/hcp-young-adult).
Behavioral performance measures	Ratings related to movie watching was collected in the original dataset (Hanke et al., 2016), which was irrelevant for the current study. The HCP dataset has various behavioral measures, which were also irrelevant for the current study.

Acquisition

Imaging type(s)	T1w and T2w structural scans. Functional scans for the StudyForrest dataset, the Raiders and Budapest datasets, and the HCP dataset.
Field strength	3 T (most data) and 7 T (HCP movie).
Sequence & imaging parameters	All structural scans used in the analysis have a spatial resolution of 1 mm or less in all directions. Detailed imaging parameters varied by dataset.
Area of acquisition	The structural and functional scans covered the entire cerebral cortex.

Diffusion MRI ☐ Used ☒ Not used

Preprocessing

Preprocessing software	We preprocessed all structural data using fMRIPrep 21.0.1 with the `--anat-only` option. The StudyForrest data were preprocessed with fMRIPrep 21.0.2. The Raiders and Budapest datasets were preprocessed with fMRIPrep 20.2.7. We used the minimally preprocessed data of the HCP dataset and removed the noise (See "Noise and artifact removal" below).
Normalization	Surface-based normalization was used for all participants.
Normalization template	Normalization was performed based on FreeSurfer's group statistics of folding patterns to derive "sphere.reg" for each participant. In the evaluation analysis, functional data were resampled to 3 different surface spaces for each resolution: fsaverage, fs_LR, and the newly created onavg.
Noise and artifact removal	A standard set of nuisance variables were regressed out from functional data, including: 6 motion parameters and their derivatives, 6 aCompCor components from WM and CSF, framewise displacement, and polynomial trends up to the 2nd order. For the HCP dataset, we used the average WM and CSF signals instead of the aCompCor components.
Volume censoring	The data used all have great quality, and we did not consider volume censoring.

Statistical modeling & inference

Model type and settings	We used a leave-one-participant-out cross-validation scheme for both the between-subject MVPC analysis and the RSA analysis. For the MVPC analysis, we predicted the left out test participant's brain responses based on the other 14 participants, and we examined whether the measured and the predicted response patterns for the same time point (TR) had the highest correlation. For the RSA analysis, for each searchlight, we computed the correlation between the test participant's RDM and the average RDM of other participants. This is the inter-subject correlation (ISC) of the RDMs, which we referred to as RSA-ISC.
Effect(s) tested	Our study compared MVPA performance based on different templates, specifically, between onavg and fsavg, and between onavg and fslr. We compared the accuracy for multivariate pattern classification and the inter-subject correlation of representational geometry. We observed that the onavg template outperformed other surface templates in all 15 participants, corresponding to a P-value of 3e-5 based on binomial testing. We reported the statistics for parametric testing in the main text.

Specify type of analysis: ☐ Whole brain ☐ ROI-based ☒ Both

Anatomical location(s)	The MVPC analysis was performed using data from the entire cerebral cortex. The RSA analysis was a searchlight analysis with a searchlight radius of 20 mm.
Statistic type for inference (See Eklund et al. 2016)	The statistics were either classification accuracy based on the entire cerebral cortex, or average RSA-ISC across all searchlights.
Correction	This study does not involve multiple comparisons across brain regions.

Models & analysis

n/a	Involved in the study
☐	☒ Functional and/or effective connectivity
☒	☐ Graph analysis
☐	☒ Multivariate modeling or predictive analysis

Functional and/or effective connectivity	Functional connectivity used in the Supplementary Information (HCP 3 T resting-state) was based on Pearson correlation.
Multivariate modeling and predictive analysis	See "Model type and settings" section above.
